# Management of High-Pressure Injection Hand Injuries: A Multicentric, Retrospective, Observational Study

**DOI:** 10.3390/jcm8112000

**Published:** 2019-11-16

**Authors:** Ermanno Vitale, Caterina Ledda, Roberto Adani, Mario Lando, Massimo Bracci, Emanuele Cannizzaro, Luigi Tarallo, Venerando Rapisarda

**Affiliations:** 1 Occupational Medicine, Department of Clinical and Experimental Medicine, University of Catania, 95123 Catania, Italy; ermannovitale@gmail.com (E.V.); cledda@unict.it (C.L.); 2 Department of Hand Surgery, Modena Polyclinic Hospital, 41124 Modena, Italy; adani.roberto@policlinico.mo.it (R.A.); lando.mario@policlinico.mo.it (M.L.); 3 Department of Clinical and Molecular Sciences, Polytechnic University of Marche, 60020 Ancona, Italy; m.bracci@univpm.it; 4 Department of Sciences for Health Promotion and Mother and Child Care “Giuseppe D’Alessandro”, University of Palermo, 90127 Palermo, Italy; emanuele.cannizzaro@unipa.it; 5 Orthopedic Department, University of Modena and Reggio Emilia, 41124 Modena, Italy; luigi.tarallo@unimore.it

**Keywords:** hand injuries, high-pressure injection, amputation, occupational risk, early treatment

## Abstract

Hand injuries after high-pressure injection are a medical emergency. These events occur frequently in workers during industrial cleaning, painting, and lubrication, and may have devastating consequences, leading to eventual amputation and poor functional outcomes. The authors have investigated the evolution, management, and outcome. Medical records of occupational medicine units and hand surgery units were collected in order to spot the high-pressure gear accident cases. Records were analyzed by dividing the subjects into two groups: those treated within 6 h and after 6 h of the trauma. A follow-up was carried out at least 1 year after treatment; the post-treatment outcomes were assessed. Of the 71 (100%) subjects, 26 (37%) were treated ≤6 h and 45 (63%) >6 h. A total of 28% (*n* = 20) underwent amputation. In 61% of cases, accidents had occurred in the iron and steel sector. High viscosity materials with a delayed treatment beyond 6 h seemed to determine compartmental syndrome and following amputation. A significantly better outcome was reported among subjects treated ≤6 h compared to those treated >6 h, 20% (*n* = 7) versus 26% (*n* = 9), respectively. Early management of this type of injury is crucial. The results of this study may contribute to providing guidelines to occupational physicians in order to best manage this type of emergency.

## 1. Introduction

Hand injuries after high-pressure injection are a rare event that can have devastating effects due to the introduction of foreign material [1]. The impact of these lesions is often underestimated [2]; indeed, right after the trauma, the small size of skin lesions (1–7 mm), the slightly perceived pain, and the reduced loss of function do not allow the right perception of the damage [1]. High-pressure injection injuries occur when equipment capable of achieving pressures sufficient to breach the human skin injects its contents into the human body [3,4]. The first reported cases described by Hesse and Rees [5,6] involved diesel fuel. Nowadays, although it is not a frequent event, it seems to be a very serious disabling condition [2]. The injury is caused by high-pressure guns emitting jet streams at pressures of 600–12,000 pounds/square inch (psi) [5]. Such high pressures bring about skin lesions that cause the shot material to get through into underlying tissues. This causes the foreign material to spread into fascial planes, tendon sheaths, and neuro-vascular planes, damaging these structures [7]. The most frequently affected hand is the non-dominant one and in particular, the index finger [8,9]. Averagely, one case on 600 hand traumatisms are due to high-pressure injection injuries [1]. Most hand injuries are work-related [10,11]. The main categories of workers affected by this type of trauma are painters, mechanics, iron and steel and naval workers, construction workers, and cattle farmers [12].

The most commonly sprayed substances are oil and paint, although cases of injection of chemical solvents, plastic, silicone, water, wax, sand, and compressed gases have also been reported [13]. The injury process is multifactorial and may be divided into four phases: initial mechanical injury caused by substance penetration [2]; chemical irritation, depending on the intrinsic cytotoxic properties of the injected substances, which cause tissue necrosis and onset of the inflammatory phenomenon [14,15]; inflammation with development of granulomatous reaction [16]; and finally, a secondary infection that is enhanced by necrosis and ischemia [5]. However, the number of infections is low, as the inorganic material, which normally penetrates the skin, does not foster bacterial proliferation [3,17].

The aim of the present study was to investigate the evolution, management, and outcome of these injuries so as to provide guidelines to occupational physicians to follow when dealing with this type of emergency.

## 2. Materials and Methods

### 2.1. Subjects

Between October 2018 and February 2019, the medical records of Catania and Ancona Universities Occupational Medicine Units as well as Modena and Reggio Emilia University Hand Surgery Units were collected. Inclusion criterion was a high-pressure gun injection injury occurring in higher limbs in the workplace. For each case selected, anthropometric parameters, professional records, and remote and recent pathological records were reported, with particular reference to the traumatic event in the workplace.

Each identified subject was contacted again for follow-up after at least one year from injury treatment. All participants provided written informed consent. During the control check, all subjects underwent an objective osteo-articular exam in order to ascertain the level of residual joint functionality (RJF), with a score ranging from 0 (joint ankylosis) to 5 (100% preserved joint mobility); the visual analogic scale (VAS), to measure the level of residual pain ranging from 0 (lack of pain) to 10 (maximum pain) [18]; and the sensitivity test according to Semmes–Weinstein, to identify any deficit in sensitivity threshold ranging from 1 (preserved sensitivity) to 20 (total loss of sensitivity).

For each patient, the outcome was assessed positive if RJF ≥2, VAS ≤5, and Semmes–Weinstein test ≤11; or negative if RJF <2, VAS >5; Semmes–Weinstein test >11. Patients with a hand or part of it amputated were ruled out of this assessment.

### 2.2. Statistical Analysis

All datasets were analyzed using SPSS version 22.0 (IBM Corp., Armonk, NY, USA). Normality was checked by Kolmogorov–Smirnov test and homogeneity of variance by Levene’s test. Descriptive analyses were performed using the percentages of frequencies. Data were also reported as a mean and standard deviation of mean. For bivariate analysis, chi-square test (χ^2^) was used to evaluate differences in categorical variables. The difference between two means was tested with unpaired Student’s *t*-test. The level of significance was set at *p* ≤ 0.05

## 3. Results

A total of 71 (100%) high-pressure injection injuries were observed, representing 2% (3550) of all cases of hand injuries occurs. The subjects examined were empirically divided into two groups according to treatment timing, within 6 h (≤6 h) (early) and after 6 h (>6 h) (delayed) from accident, in accordance with Hogan and Ruland [8], in which the risk of amputation decreased if a thorough surgical debridement was carried out within 6 h of the accident. Of the subjects, 68 (96%) were males, and their mean age was 39.4 ± 10.3 years. Duration of employment was 17.2 ± 5.5 years. All had been assigned to the same job for at least 5 years. For each subject, the first treatment had been given at the first aid unit of the nearest hospital. Table 1 reports the main features of the sample divided in two subgroups.

Of the 71 patients, 26 (37%) were treated within ≤6 h (4.1 ± 1.8 h) from trauma and 45 (63%) after >6 h (12.94 ± 8.1 h). Their occupation was as follows: 43 (61%) were employed in the iron and steel industry, 13 (18%) in naval painting firms, 7 (10%) were construction workers, 6 (9%) farmers, 1 (1%) truck drivers, 1 (1%) lumberjack. From data analysis it was observed that in 61% (*n* = 43) of cases, accidents had occurred in the iron and steel sector; a significant number (*n* = 32) of these workers had been treated after 6 h, whereas in accidents occurring in other sectors, no statistically significant difference was observed.

All workers were right-handed; 55% (*n* = 39) of lesions involved the left hand (non-dominant) and 45% (*n* = 32) the right hand. No bilateral lesion was recorded. Table 2 reports the trauma localization and the injected material.

In 36 (51%) cases, the index finger was injured, in 7 (10%) the thumb, in 4 (6%) the middle finger, in 2 (2%) cases the 4th finger, in 1 (1%) case the 5th, in 3 (4%) cases more fingers were involved, and in 18 (26%) the palm of the hand was involved. 

A statistically significant increase was detected of lesions to the index finger (*n* = 36) of which 60% (*n* = 27) had been treated >6 h. The main substances injected were as follows: 40 (57%) hydraulic oil, 10 (14%) industrial grease, 8 (12%) epoxy paint, 4 (5%) paint solvent, 5 (7%) trichlorethylene, 1 (1%) mercury, 1 (1%) glue, 1 (1%) propylene oxide, and 1 (1%) epoxy stucco.

Hydraulic oil was the most statistically significant injected material in these kinds of injuries.

Early clinical manifestations had been characterized in all cases by few symptoms, slight pain, and limited reduction of joint functionality. After about 2–3 h, pronounced pain and edema with skin inflammation involved the whole hand.

Once at the first-aid unit, all patients initially received anti-inflammatory drugs (non-steroidal anti-inflammatory drugs or corticosteroids) if deemed necessary, as well as anticoagulants, broad-spectrum antibiotics, and tetanus toxoid, unless previously vaccinated. All subjects underwent surgical treatment consisting of decompression, debridement, drainage, and, in most serious cases, partial or total amputation. Amputation was needed in 20 (28%) subjects, 5 (7%) among patients treated ≤6 h and 15 (21%) among patients treated >6 h. Table 3 shows the main characteristics of amputation-ending traumas.

The number of subjects treated within 6 h who underwent amputation was significantly lesser than that of patients who had been amputated but after 6 h (5 vs. 15 subjects).

Analysing the amputation causes, 2 (10%) were after lesions to internal tissues, directly caused by mechanical trauma, impossible to repair, namely caused by hydraulic oil (*n* = 1) and industrial grease (*n* = 1); 7 (35%) were from a chemical reaction, with tissues damaged due to intrinsic cytotoxic action of the injected substances, in particular paint solvents (*n* = 4), epoxy paint (*n* = 2), and trichlorethylene (*n* = 1); 5 (25%) were due to tissue ischemia with following necrosis, in particular hydraulic oil (*n* = 5); and 6 (30%) were from onset of compartmental syndrome, in particular industrial grease (*n* = 4), epoxy stucco (*n* = 1), and glue (*n* = 1). No cases of infection were reported. Among subjects treated within 6 h, compartmental syndrome was the most frequent cause of amputation.

In 10 (50%) cases the index finger was amputated, in 3 (15%) cases the thumb, in 1 (5%) case the middle finger, in 2 (10%) cases the 4th finger, in 1 (5%) case the 5th finger, and in 3 (15%) cases more fingers were amputated.

The substances injected were hydraulic oil in 6 (30%) cases, in 5 (25%) industrial grease, in 4 (20%) paint solvent, in 2 (10%) epoxy paint, in 1 (5%) trichlorethylene, in 1 (5%) glue, and in 1 (5%) epoxy stucco. Hydraulic oil was the substance most involved among subjects that underwent amputation.

Factors that delayed intervention (>6 h) were as follows: 78% (*n* = 35) of cases were poor early symptoms; 16% (*n* = 7) were from difficulty reaching the closest first-aid unit quickly; and 6% (*n* = 3) were from underestimation of the event by the first-aid unit healthcare staff who gave a yellow code in the triage.

Of the 71 (100%) subjects, 59% (*n* = 42) turned up for follow-up. Among them, 17 (40%) had been treated within 6 h and 25 (60%) after the accident, whereas, of the 29 (41%) who did not turn up at follow-up, 9 (31%) had been treated within 6 h and 20 (69%) more than 6 h after the accident.

A total of 29 (100%) subjects did not undergo any follow-up: 16 (8%) did not answer the request (12 of which were among the amputated patients), 10 (7%) said they were not interested, and 3 (1%) set the check date but never turned up.

In assessing the outcomes and ruling out the amputated subjects (another 8 subjects), 15 patients were left among those treated ≤6 h and 19 treated after (Table 4).

A better but not significant outcome was reported in the group of subjects treated within 6 h from the accident compared to those treated after. In detail, in the former group, the positive outcome was observed in nine (26%) subjects treated within 6 h versus seven (20%) subjects treated after.

Analysing the scores of the evaluation scale used, a significant reduction of pain symptoms can be observed (VAS scale), as well as a better residual joint functionality (RJF) in subjects treated within 6 h versus those treated after.

Analysing the 5 (25%) amputated patients treated ≤6 h and the 15 (75%) treated after 6 h also with amputation and considering the substances injected, the following rates were observed: 15% (*n* = 6/40) hydraulic oil, 50% (*n* = 5/10) industrial grease, 25% (*n* = 2/8) epoxy paint, 100% (*n* = 4/4) paint solvent, 20% (*n* = 1/5) trichlorethylene, 100% (*n* = 1/1) mercury, 100% (*n* = 1/1) glue, and 100% (*n* = 1/1) epoxy stucco.

## 4. Discussion

Despite being a rare event, injuries to the hand caused by high pressure injection devices represent a major medical emergency [2]. These devices exploit the energy potential of the air emitted by a pump which can penetrate the skin and underlying structures [2]. 

In our experience, these specific injuries stood for 2% of all traumatic lesions in workplaces occurring over the last 15 years. This is positive data as it only regards a few cases compared to the large presence of these instruments in several manufacturing areas such as agriculture, industry, and maintenance services in general [3,10,11,12].

In 61% (*n* = 43) of cases, accidents had occurred mainly in the iron and steel sector; these data are attributable to a high number of both industrial and craftsman workplaces spread in our territory [19,20,21].

High-pressure injection injuries occur when equipment capable of achieving pressures sufficient to breach the human skin injects its contents into the human body [3,6].

When the limb is penetrated, the material spreads into tissues and rapidly invades them. The diffusion through tissues depends on the material density and the shooting speed, apart from resistance the tissues of interest offer [22,23].

Usually, the impact of these lesions is often underestimated, owing to the small entry size and few symptoms; however, within a few hours, symptoms become more important, with strong pain, swelling, and loss of hand functionality [1,2].

Immediate surgical exploration and debridement with meticulous removal of the injected material has been shown as the most appropriate treatment [8,24,25]. Debridement of all necrotic tissue is crucial before any attempt at reconstruction [26].

In the present study, in all 71 subjects, symptoms after the trauma had been few, with slight pain and limited reduction of joint functionality. However, after about 2–3 h pronounced pain and edema with skin inflammation involved the whole hand. Despite the increasing symptoms, only 37% (*n* = 26) of subjects were treated within ≤6 h from trauma. 

Literature data highlights how the early hours are the most crucial in trauma treatment, after which the need for amputation increases significantly [27]. For this reason, we analyzed our sample by dividing it into two groups according to treatment timing (within or after 6 h). Of the 71 (100%) subjects under exam, 63% (*n* = 45) had been treated after 6 h from trauma, in an average time of 12.94 ± 8.1 h.

A total of 55% (*n* = 39) of lesions involved the non-dominant hand (left hand) and, in particular, the index finger (*n* = 36).

These data are in line with what has been reported in the scientific literature, wherein the most frequently affected hand is the non-dominant one and, in particular, the index finger [8,9].

The substance injected with significantly greater frequency, compared to others, was hydraulic oil (57%). This observation is accounted for by the high number of cases that have been observed in the iron and steel sector, where hydraulic oil (57%) and industrial grease (14%) are widely employed.

In this study, we reported a statistically significant increase of the number of lesions that involved the non-dominant hand’s index finger, which is in line with what is reported in the literature [2,6], probably because the non-dominant hand index finger is placed close to the target in order to stabilize it [5].

The work activity mostly involved in our study was the iron and steel sector. In accordance with the literature, injuries occur while greasing gears and cleaning nozzles. Often, many of these injuries involve an employee of a new job or handling unfamiliar equipment [1,9,28]. In our sample, all workers had a multi-annual work experience. As described by Garus-Pakowska et al. [29] and by Thepaksorn et al. [30], daily work in the same environment and the same tools generate familiarity towards places and objects, which makes them erroneously lower the attention level, thus increasing injury risks. 

All subjects were treated surgically, but only 37% (*n* = 26) within 6 h from injury. In particular, the initial treatment had involved drugs: anti-inflammatory drugs (non-steroidal anti-inflammatory drugs or corticosteroids) if deemed necessary, as well as anticoagulants, broad-spectrum antibiotics, and tetanus toxoid, unless previously vaccinated [20,31]. Later, subjects underwent surgical treatment consisting of decompression, debridement, drainage, and, in most serious cases, partial or total amputation [32].

The amputation was carried out in 20 (28%) subjects, 5 (7%) among patients treated ≤6 h, and 15 (21%) among patients treated >6 h after injury. Hogan and Ruland [8], in a literature review of 435 cases of high pressure injection injuries, showed that the overall risk of amputation was 30%. In our study, the main causes were found to be lesions of internal tissues in 10% of cases, caused by the mechanical trauma, impossible to be repaired; in 35% from chemical reaction, with damage to tissues due to intrinsic cytotoxicity of the injected substances; in 25% due to ischemia of tissues with following necrosis; and for the remaining 30%, with onset of compartmental syndrome.

Analyzing the causes of amputation in relation to the substance injected, it is to be pointed out that paint solvent has always (100% of cases) caused chemical reaction-related tissue damage, followed by epoxy paint and trichlorethylene. Aside from this, compartmental syndrome has been observed in patients treated after 6 h, when high viscosity substances were involved, such as industrial grease, epoxy stucco, and glue. Instead, tissue ischemia with following necrosis was determined by hydraulic oil, especially in subjects treated later. These data show that some substances such as paint solvent lead to a worse outcome, as they cause tissue damage due to the chemical reaction. Substances with high viscosity, such as industrial grease, epoxy stucco, glue, and hydraulic oil, lead to an unfavorable outcome probably due to the action combined with the delay (>6 h) in the treatment.

In a follow-up, only 59% (*n* = 42) of patients were checked. The authors assessed the outcome after at least 1 year, checking residual pain (VAS), residual joint functionality (RJF), and sensitivity threshold (Semmes–Weinstein). A significantly better outcome was reported in the group of subjects treated within 6 h from the accident compared to those treated after, as far as residual pain is concerned (VAS). Amputation and/or loss of joint functionality are considered as negative outcomes [5]. In particular, among those who came for follow-up, 40% (*n* = 6) of patients treated ≤6 h showed negative outcomes, whereas 63% (*n* = 12) of those treated >6 h showed negative outcomes. 

Analysing the number of amputated subjects, it is possible to observe that this was significantly lower in those who had been treated within 6 h compared to those treated after (5 vs. 15 subjects). Therefore, it is confirmed that the treatment time seems to be decisive in the outcome of this type of injury.

Schoo et al. [21] have shown that the most important factor in determining the outcome was the material injected, reporting an overall amputation rate of 48%. They reported an amputation rate of 80% for turpentine (resin) injections, 58% for paint injections, and 20% for grease injection. In the same way, a literature review from 1966 to 2003 by Hogan and Ruland [8] also demonstrated that the material injected was a significant factor in determining the risk of amputation. Amputation was required in >40% of cases involving organic solvents, including paint thinner, paint, diesel fuel, gasoline, jet fuel, and oil. Aside from this, in a retrospective study by Mirzayan et al. [33] on 35 patients, a correlation between paint type and amputation risk was observed.

In our sample, according to the literature, several types of materials were detected [13,34]; no resins were found. Our data confirm that solvents and paints provoke chemical reaction in tissues leading to tissue damage and amputation.

Furthermore, it seems that high viscosity substances such as industrial grease, epoxy stucco, and glue, together with a delayed treatment beyond 6 h, may foster the onset of compartmental syndrome and following amputation. 

Among the cause for amputation, we detected a statistically significant increase of amputated subjects after 6 h 100% (*n* = 6) after onset of compartmental syndrome—the high pressure of the substance inside limited compartmental volumes and acute inflammatory reaction, causing the compartmental pressure to rise, which generates vase spasms, bringing further swelling, local ischemia, and thrombosis. The outcome of this always increasing inflammatory response is a vicious circle of swelling, ischemia, and, eventually, compartmental syndrome, especially when more viscous substances are involved. [1,35]. On the other hand, ischemia of tissues with following necrosis was caused by hydraulic oil, especially in subjects who were treated later than necessary.

The literature data show how the injection of non-toxic substances such as air or water give better outcomes and do not end up with any amputation, probably owing to the innocuous nature of these materials [1].

In accordance with literature data, early surgical debridement is critical for controlling the inflammatory response, decompressing the compartments, and reducing the risk of long-term morbidity and amputation [8,35,36]. Delays in surgery have been reported by several authors to have a higher incidence of morbidity and amputation [2,14].

In our study, we observed that the main reason in delaying treatment was the underestimation of the trauma on the worker’s part, in line with literature data [1,2,8]; this is due to the small size of the wound and scarcity of symptoms, which reduce the risk awareness. Furthermore, this underestimation also occurred in 3 (6%) cases with triage first-aid unit healthcare personnel. As a matter of fact, literature data highlight how the early hours are the most crucial in trauma treatment, after which the need for amputation increases significantly [27].

Bashir et al., (2017) [37] reported this correlation, showing an increase of the amputated subjects who had been treated after 10 h from injury. We found no strict correlation between the time (hours) of treatment after trauma and its outcome. Instead, it seems that it is crucial to start the first interventions within 6 h compared to after if the risk of compartmental syndrome is to be reduced.

This study showed a better outcome in those subjects treated precociously compared to those treated after 6 h.

At the follow-up, a significantly better VAS was detected in the subjects treated ≤6 h from an accident. This could be put down to an early treatment of the phlogosis process caused by the material and the probable complications such as necrosis [37]. 

Limitations of this study were the lack of information about the injection pressures into tissues, as higher pressure levels may cause worse outcomes [2]. Moreover, only 42 (59%) patients showed up for follow-up, which may have been due to the lack of confidence in the medical center where they were treated or lack of interest in the medical examination due to good general clinical condition. In the sample considered, non-toxic material injections were not taken into account, such as air or water, which normally end up with better outcomes [1]; furthermore, the small number of the sample and the variety of substances reduce the possibility of obtaining definitive results. 

## 5. Conclusions

High pressure lesions are rare clinical events that often leave invalidating aftermaths. From a comparison of our data with those already in the literature, it is clear how the time elapsing between the trauma and treatment is crucial in determining a better outcome, especially in relation to the type of material injected. As reported in the literature, delaying surgical intervention increases the incidence of morbidity and amputation [8,24]. This should draw attention on the management of early treatment phases in the workplace. In order to do this, it is essential to instruct workers so that they do not underestimate the importance of the trauma. This may bring about a reduction in the number of amputations in workers at risk and would result in better functional results.

## Figures and Tables

**Table 1:** Main sample characteristics divided according to treatment timing (≤6 h and >6 h).

	Treatment Timing		
Subjects	26 (37%) ≤6 h	45 (63%) >6 h	*p*-Values
Age (years)	39.1 ± 11.0	39.5 ± 10.1	n.s.
Male (%)	26 (37%)	42 (59%)	n.s
Female (%)	0	3 (4%)	n.s.
Duration of employment (years)	17.4 ± 5.7	18.1 ± 5.2	n.s.
Employment in the same area (years)	9.6 ± 6.1	10.1 ± 7.0	n.s.
Iron and steel industry	11 (16%)	32 (45%)	<0.05
Naval painting	6 (8%)	7 (10%)	n.s.
Construction workers	4 (6%)	3 (4%)	n.s.
Farmers	4 (6%)	2 (3%)	n.s.
Truck drivers	0	1 (1%)	n.s.
Lumberjack	1 (1%)	0	n.s.

n.s.: not significant.

**Table 2:** Trauma localization and injected material divided according to treatment timing.

		Treatment Timing		
		26 (37%) ≤6 h	45 (63%) >6 h	*p*-Values
**Hand Injured**	Right hand injuries	11 (16%)	21 (29%)	n.s.
	Left hand injuries	15 (21%)	24 (34%)	n.s.
	Bilateral injury	0	0	n.s.
**Hand Parts Affected**	1st finger	4 (6%)	3 (4%)	n.s.
	2nd finger	9 (13%)	27 (38%)	^^,^*<0.05
	3rd finger	2 (3%)	2 (3%)	n.s.
	4th finger	1 (1%)	1 (1%)	n.s.
	5th finger	0	1 (1%)	n.s.
	More fingers	0	3 (4%)	n.s.
	Palm	10 (14%)	8 (12%)	n.s.
**Injected Material**	Hydraulic oil	15 (22%)	25 (36%)	°<0.05
	Epoxy Paint	4 (6%)	4 (6%)	n.s.
	Paint solvent	3 (4%)	1 (1%)	n.s.
	Industrial grease	3 (4%)	7 (10%)	n.s.
	Trichlorethylene	0	5 (7%)	n.s.
	Glue	0	1 (1%)	n.s.
	Mercury	0	1 (1%)	n.s.
	Propylene oxide	1 (1%)	0	n.s.
	Epoxy stucco	0	1 (1%)	n.s.

n.s.: not significant; ^ subjects treated ≤6 h versus subjects treated >6 h; * hand parts affected; ° injected materials.

**Table 3:** Characteristics of amputation ending traumas.

Variables		≤6 h 5 (25%)	>6 h 15 (75%)	*p*-Values
**Causes of Amputation**	Mechanical trauma	1 (5%)	1 (5%)	n.s.
	Chemical reaction	3 (15%)	4 (20%)	n.s.
	Compartment syndrome	0	6 (30%)	^<0.05
	Ischemia and necrosis	1(5%)	4 (20%)	n.s.
	Infection	0	0	n.s.
**Hand Parts Amputated**	1st finger	1 (5%)	2 (10%)	n.s.
	2nd finger	3 (15%)	7 (35%)	n.s.
	3rd finger		1 (5%)	n.s.
	4th finger	1 (5%)	1 (5%)	n.s.
	5th finger		1 (5%)	n.s.
	More fingers		3 (15%)	n.s.
**Injected Material**	Hydraulic oil	0	6 (30%)	^<0.05
	Epoxy paint	0	2 (10%)	n.s.
	Paint solvent	3 (15%)	1 (5%)	n.s.
	Industrial grease	2 (10%)	3 (15%)	n.s.
	Trichlorethylene	0	1 (5%)	n.s.
	Glue	0	1 (5%)	n.s.
	Epoxy stucco	0	1 (5%)	n.s.
**Total of Amputees**		**5 (25%)**	**15 (75%)**	^<0.05

n.s.: not significant; ^ subjects treated ≤6 h versus subjects treated >6 h.

**Table 4:** Follow-up results on 34 subjects.

Subjects/Treatment Time	15(44%)/≤6 h	19(56%)/>6 h	*p*-Values
Visual analogic scale (VAS) ≤5	13 (38%)	9 (26%)	**<0.05**
Residual joint functionality (RJF) ≥2	9 (21%)	7 (20%)	n.s.
Semmes–Weinstein ≤11	10 (29%)	10 (29%)	n.s.
**Positive outcome**	**9 (26%)**	**7 (20%)**	**n.s.**

n.s.: not significant.
